# Jujube and green tea extracts protect human fibroblast cells against UVB-mediated photo damage and MMP-2 and MMP-9 production

**Published:** 2020

**Authors:** Zahra Abotorabi, Mohsen Khorashadizadeh, Mina Arab, Mohammad Hassanpour Fard, Asghar Zarban

**Affiliations:** 1 *Clinical Biochemistry Department, Faculty of Medicine,Birjand University of Medical Sciences, Birjand, Iran*; 2 *Cellular and Molecular Research Center, Birjand University of Medical Sciences, Birjand, Iran*; 3 *Department of Medical biotechnology, Faculty of Medicine, Birjand University of Medical Sciences, Birjand, Iran*; 4 *Pharmacology Department, Faculty of Medicine, Birjand University of Medical Sciences, Birjand, Iran*

**Keywords:** UVB, Jujube, Green tea, Oxidative stress, MMPs

## Abstract

**Objective:**

Oxidative stress and ultraviolet B (UVB) irradiation are known as principal inducers of DNA damage and modulators of gene expression in aging process and skin photoaging, which are associated with upregulation of matrix metalloproteinases (MMPs). Because of the antioxidant capacity of jujube and green tea, we decided to determine their protective effects of human fibroblast cells against UVB-induced photo-damage and reduction of MMP-2 and MMP-9 expression.

**Materials and Methods:**

We exposed human fibroblast cells to different doses of UVB (0-20 mJ/cm^2^) with or without different concentrations of jujube and green tea extracts. Cell viability was assessed using MTT assay. Total antioxidant capacity and free radical scavenging activity of cell supernatant were assessed using FRAP and DPPH methods, respectively. The concentrations of MMP-2 and MMP-9 in the samples were determined by ELISA kits.

**Results:**

Fibroblast cells viability, 24 hr after UVB irradiation, reduced about 70% compared to the controls. Pre-treatment of the cells with jujube extract (8 mg/ml) increased the cell viability by almost 85% while green tea (0.5 mg/ml) protected the irradiated cells by 71%. Also, MMP-2 and MMP-9 content decreased in a concentration-dependent manner in the cells pre-treated with jujube and green tea extracts.

**Conclusion:**

These data suggest that jujube and green tea could be useful to attenuate solar UVB light-induced oxidative stress and skin photoaging and can be suggested as a potential candidate for the development of new anti-UVB medicines and cosmetic products.

## Introduction

The sun is the major source of UV radiation. UV radiation which exists in the sunlight can be categorized into three major groups including UVA (320-400nm), UVB (290-320nm), and UVC (<290nm). In this regard, UVA and UVB reach the earth surface and can have harmful effects on human health. UVB has more harmful effects on living creatures because of the higher energy it possesses (Azuha et al., 2013 ▶; Zegarska et al., 2016 ▶). UVB can lead to damage directly or indirectly. For example, it can be directly absorbed by DNA base and lead to formation of dimers between pyrimidine existing in a DNA chain, particularly thymine dimer which eventually leads to the carcinogenic C-T mutation. Also, it can cause some damage indirectly via induction of oxidative stress (Duan et al., 2019 ▶). Many studies indicated that exposure of the skin to UV radiation leads to formation of active oxygen and nitrogen species followed by subsequent oxidative stress (Synowiec et al., 2015 ▶).

In fact, oxidative stress is defined as the imbalance between anti-oxidant defense system of the body and generation of active oxygen species leading to cell damage, cell death, and severe age-related diseases such as cancers, cardio-vascular diseases and different kinds of neurodegenerative diseases (Farajdokht et al., 2017 ▶)

Besides, UVB activates proteinase enzymes, particularly matrix metalloproteinase (MMPs). MMPs are a family of proteolytic enzymes which can play an important role in digestion of many extra-cellular matrix compounds as well as basic membrane tissue, and are accordingly important in biological and pathological processes. Long-time UVB exposure causes alterations in the extra-cellular matrix by production of MMPs which can accelerate photoaging (Hibbert et al., 2018 ▶).

In recent years, medicinal plants with antioxidant features were extensively studied to deal with oxidative stress (Yamagishi et al., 2008 ▶). Moreover, there is a direct relationship between the antioxidant activity and content of polyphenolic compounds in plants (Hemmati et al., 2015 ▶). Jujube is a flowering plant from dicotyledonous category, Rosales order, Rhamnaceae family, *Ziziphus *genus, and *Ziziphus jujube *species. The trees usually grow in dry and semi-arid regions of Iran, particularly South Khorasan. It can also be found in wide areas of Asia, Africa, and South America and its scientific name is *Ziziphus jujube *(Al- Reza et al., 2009 ▶).

Investigations have revealed that jujube contains many active biological compounds such as alpha-tocopherol, flavonoids, beta-carotene, phenolic acids and polysaccharides types with inhibitory effects on histamine release, cyclooxygenase I and II and choline esterase activation. It has also cytotoxic effects on cancer cells through alteration of some apoptotic genes expression (Hoshyar et al., 2015 ▶). This plant is used in the treatment of many diseases such as allergy, urinary problems, and chronic bronchitis, constipation, and sleep disorders (Li et al., 2007 ▶).

Green tea is obtained from the leaves of Camellia sinensis and contains polyphenols, flavonoids, carotenoids, glycoproteins, caffeine, catechins, fiber, lipids and vitamins B and C. Green tea and its main component, catechin has been specifically considered for antioxidant features. Studies performed in animal models, indicated that green tea has protective effects on different kinds of cancer such as breast, skin, lung and prostate cancer (Williamson et al., 2011 ▶; Naponelli et al., 2017 ▶). Moreover, studies showed that compounds found in green tea can reduce the level of MMPs, especially MMP-2 and MMP-9 (Benelli et al., 2002 ▶).

Given the features of jujube and green tea, we assumed that consumption of these herbs can reduce skin aging markers via inhibition of oxidative damages induced by UV radiation and reduction of MMPs proteins. In the present study, oxidative damages induced by UVB radiation in human normal fibroblast cells, along with protective effects of jujube and green tea extracts against these damages, were studied.

## Materials and Methods

**Chemicals and reagents**

Dulbecco’s modified eagle media (DMEM), fetal bovine serum (FBS), antibiotics, and trypsin-EDTA were obtained from BioSera Co. (France). 3-(4, 5-dimethylthiazol-2-yl)-2, 5-diphenyltetrazolium bromide (MTT) and dimethyl sulfoxide (DMSO) were purchased from Sigma-Aldrich chemical (St. Louis, USA). 2, 2-diphenyl-1-picrylhydrazyl (DPPH) and 2, 4, 6-tri (2-pyridyl)-s-triazine (TPTZ) for spectrophotometry (det. P99.0%) were purchased from Fluka (Buchs, Switzerland). HCl, iron (III) chloride (FeCl_3_), iron (II) sulfate heptahydrate (FeSO_4_.7H_2_O) were purchased from Merck Chemicals (Darmstadt, Germany). MMPs ELISA kits were obtained from Abcam (Cambridge MA, USA).

**Preparation of herbal boiled extract**

Jujube was collected from farms of South Khorasan (Voucher sp. No.: E 1147-FUMH), Iran. Green tea was purchased from a grocery/market. In order to prepare aqueous extracts, 5 g of dried and crushed powder of each sample was added to 100 ml of boiling water. After 10 min, the mixtures were filtered through Whatman No.1 filter paper and the samples were lyophilized. To prepare different concentrations of jujube and green tea, the dried extracts were diluted in DMEM cell culture medium.

**Cell culture and UVB irradiation**

The normal human fibroblast cells were received from the cell bank of Stem Cell Research Center (Tehran, Iran) as a gift. The cells were cultured in complete DMEM medium containing 10% FBS, 100 unit/ml penicillin, and 100 mg/ml streptomycin and grown at 37°C in humidified atmosphere containing 5% CO_2_. For UVB irradiation, fibroblast cells were seeded at a density of 1×10^4 ^cells in 2ml complete DMEM in 35mm plates and left overnight to attach; on the next day, UVB treatment was done.

**MTT assay**

In order to evaluate the cytotoxicity of UVB on fibroblast cells, different doses of UVB (0-20 mJ/cm^2^) were applied. Different concentrations of the extracts (1-8 mg/ml) were also used to determine their effects on fibroblast cell proliferation. After 24 hr, cell viability was measured by 3-(4, 5- dimethyl-2-thiazolyl)-2, 5-diphenyl-2H-tetrazoliumbromide (MTT) assay (Mosmann, 1983). To each 35mm plates, 200 µl of MTT (5 mg/ml) reagent was added and incubated for the next 4 hr. Thereafter, the supernatant was removed, and any precipitate present was dissolved by adding 500 µl of DMSO. The absorbance was read at 540 nm against 620 nm using a plate reader (Epoch, Biotek USA).

**Determination of total antioxidant capacity of cell culture media**

In order to measure the total antioxidant capacity of the cell culture media with or without herbal extracts, ferric reducing antioxidant power (FRAP) assay was performed for the supernatants of cell cultures. The assay is based on the reduction of ferric to ferrous ions at low pH, providing an intense blue color using 2, 4, 6-tripyridyl-s-triazine (TPTZ) reagent. The color intensity can be monitored by measuring the absorbance at 593 nm (Benzie and Strain, 1996 ▶). Briefly, 5 µl of each sample was mixed with 200 µl of freshly prepared FRAP reagent. The solutions were incubated at 37°C for 20 min and the measurement was then carried out by a spectrophotometer plate reader (Epoch, BioTeck, USA) at 593 nm.

**DPPH radical scavenging activity**

Free radical scavenging activity in culture media of the fibroblast cells treated with UVB and/or herbal extracts, was evaluated by the DPPH method. Briefly, 20 µl of each sample was added to 200 µl of DPPH reagent. After incubation for 10 min at room temperature, the absorbance of the solution was determined at 517 nm by a spectrophotometer plate reader (Epoch, BioTeck, USA).

The scavenging ability of DPPH was calculated according to the following equation:

Scavenging activity (%) = [(absorbance of the control – absorbance of the sample)/absorbance of the control]* 100

**Evaluation of MMPs using the enzyme-linked immunosorbent assay (ELISA)**

To evaluate MMP-2 and MMP-9 secretion into the culture media, fibroblast cells were seeded in 35 mm plates at a density of 1×10^5 ^in 2 ml DMEM medium containing 1% FBS, and then, incubated overnight at 37°C. Before treatment of the cells with UVB and the extracts, the cells were washed twice with 1 ml of PBS. Then, in the presence of fresh PBS, the cells were exposed to UVB at 4 mJ/cm^2^. PBS was then removed and the extracts were added. The supernatants of cell culture were collected after 24 hr. The MMP-2 and -9 concentrations in the cell supernatants were determined by ELISA kits according to the manufacturer’s instructions.

**Statistical analysis**

All experiments were performed in triplicate samples and repeated at least three times. The data are presented as means±SD and statistical comparisons among groups were performed using one-way ANOVA. A p<0.05 indicated significant differences.

## Results

**Effect of UVB on fibroblast cell viability**

**Figure 1:**
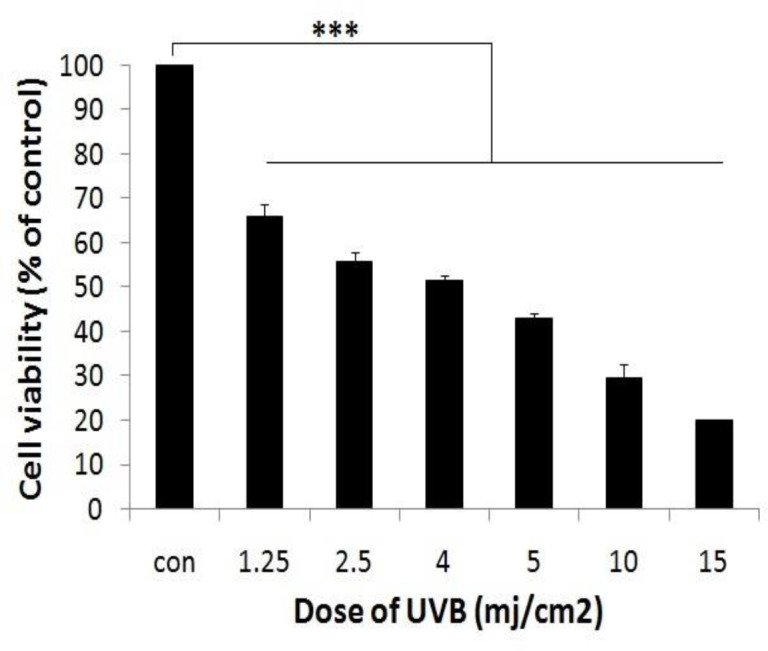
The cytotoxicity of UVB in fibroblast cells. Fibroblast cells were irradiated with different doses of UVB and after 24 hr, cell viability was determined by MTT assay). ***p<0.001, significant difference between irradiated groups when compared to the control group

The results showed that UVB treatment, even at the lowest dose of 1.25 mJ/cm^2^, reduced fibroblast cells viability to about 70% of control cells (p<0.001). Furthermore, by increasing dose of UVB, cell viability was further reduced and IC_50_ was 4 mJ/cm^2^ (Figure 1).

**Anti-UVB effects of jujube and green tea extracts in fibroblast cell**

**Figure 2:**
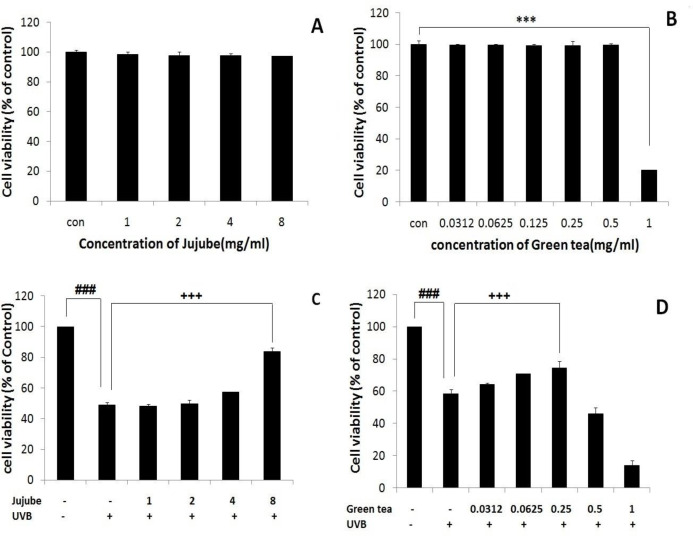
Effect of jujube and green tea extracts on fibroblast cell viability. Fibroblast cells were treated with different concentration of jujube (A) and green tea (B) extracts and after 24 hr cell, viability was determined by MTT assay. The protective effect of herbal extracts against UVB damages in fibroblast cell: The fibroblast cells were treated with jujube and green tea extract for 30 min and then, exposed to UVB irradiation at 4 mJ/cm^2^. (C) The protective effect of different concentrations of jujube on fibroblast cells. (D) The protective effect of different concentrations of green tea on fibroblast cells). ***p<0.001, significant difference between different concentrations of treated groups and the untreated control group. ###p<0.001, significant difference between irradiated group when compared to the control group. +++p<0.001, significant difference between irradiated group when compared to the concomitant irradiate and treated group

Treatment with jujube and green tea extracts alone at a concentration range of (0-8 mg/ml) and (0-1 mg/ml), respectively, did not show any reduction in fibroblast cell viability by 24 hr, but high concentrations of green tea induced cytotoxic effects in fibroblast cells and cell viability was reduced (p<0.001) (Figures 2A and 2B). In order to study the protective effects of jujube and green tea extracts in fibroblast cells against UVB irradiation, we also used MTT assay. Pre-treatment with jujube and green tea for 30 min followed by UVB irradiation and further incubation at 37°C for 24 hr, showed that, 8mg/ml of jujube extract was capable of protecting fibroblast cells, with an almost 85% increase in cell viability observed in treated fibroblast cells (p<0.001) (Figure 2C). Pre-treatment with green tea at a concentration range of 0-1mg/ml with the same manner followed by UVB irradiation, indicated that low concentrations of green tea had almost equal protective effects and increased cell viability up to approximately 71% (p<0.001) (Figure 2D).

**Evaluation of the antioxidant activity of cell culture media in the presence of jujube and green tea extracts**

**Figure 3:**
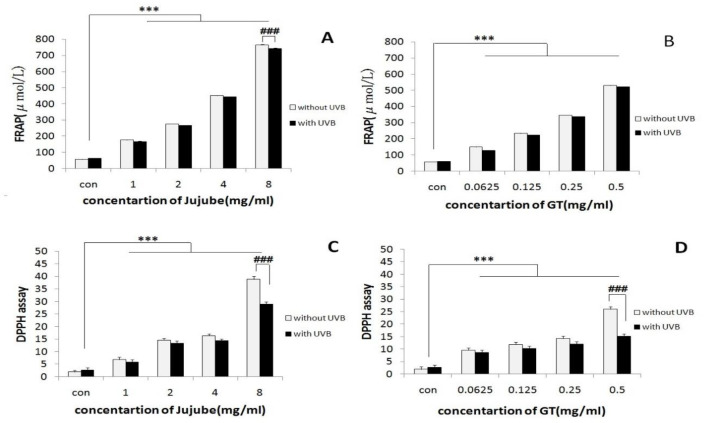
The effects of jujube (A and C) and green tea (B and D) extracts on antioxidant capacity of fibroblast cells culture media. FRAP and DPPH methods were performed on supernatant of the cells. Based on the results, the antioxidant capacity of culture media was increased by adding jujube and green tea extract, in a concentration-dependent manner. ***p<0.001, significant difference between different concentrations of treated groups and the untreated control group. ###p<0.001, significant difference between irradiated group when compared to non-irradiated group

Based on the results of both DPPH and FRAP assays, the antioxidant capacity of culture media was increased by adding both jujube and green tea extracts, in a concentration-dependent manner with or without UVB irradiation (p<0.001) (Figures 3A, 3B, 3C, and3D).

**Effect of jujube and green tea extracts on UVB-induced MMP-2 and -9 secretion in fibroblast cells culture media**

**Figure 4:**
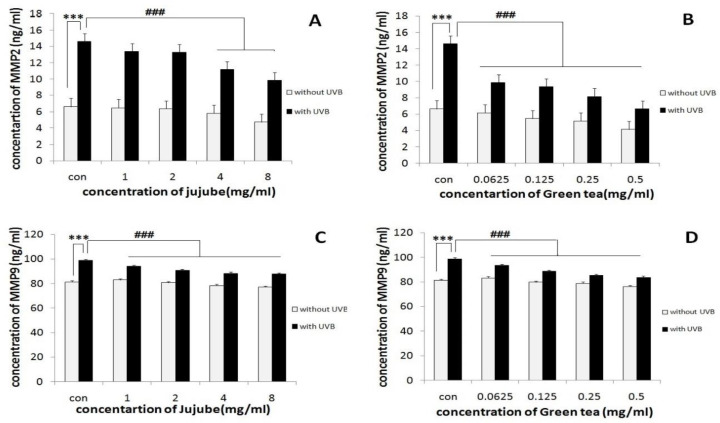
(A and C): Effect of jujube extract on UVB-induced MMP-2 and MMP-9 secretion by fibroblast cells. The cells were incubated with various concentrations of jujube for 30 min, then exposed to UVB (4 mJ/cm^2^), and incubated for the next 24 hr at 37 C. (B and D): Effect of green tea extract on UVB-induced MMP-2 and MMP-9 secretion by fibroblast cells. The cells were incubated with various concentrations of jujube for 30 min, then exposed to UVB (4 mJ/cm^2^), and incubated for the next 24 hr at 37 C. ***p<0.001, significant difference between irradiated group when compared to the control group. ###p<0.001, significant difference between irradiated group when compared to the concomitant irradiated and treated group

In the presence of UVB irradiation, the protein level of MMP-2 and -9 were significantly increased (p<0.001) (Figure 4). 

Nevertheless, treatment of the fibroblast cells with jujube and green tea extracts (Figure 4A and B), reduced MMP-2 and -9 protein in a dose-dependent manner. At low concentrations, the green tea-treated groups demonstrated notable decreases in MMP-2 and MMP-9 levels compared to the jujube-treated groups (p<0.001).

## Discussion

UV radiation is a major environmental risk factor that can lead to harmful effects through various molecular mechanisms, such as DNA damages and gene expression modulation (Zegarska et al., 2016 ▶). Some compounds with antioxidant features are produced as secondary products by plants after exposure to active oxygen species. It was shown that antioxidants can lead to reduced oxidative stress induced by UVB radiation by neutralizing ROS (Magalhaes et al., 2009 ▶).

In this study, we showed that fibroblast cells viability was decreased when exposed to UVB radiation; however, in the presence of jujube and green tea extracts, the percentage of viable cells was increased compared to control cells.

We first showed that UVB radiation significantly decreased fibroblast cells viability in a dose-dependent manner, but treatment with different concentrations of jujube and green tea were safe for the cells at determined concentrations; these results were in line with the results of other researches (Salucci et al., 2017 ▶). Anggakusuma et al. (2010) ▶ revealed that UVB radiation by increasing ROS production and changing the redox status of the cells, reduced HaCaT cell viability in a dose-dependent manner (Anggakusuma et al., 2010 ▶). Also, in our study, when fibroblast cells were pretreated with jujube and green tea, and then exposed to UVB, cell viability was significantly increased in cell line with increasing concentration of both jujube and green tea. These results indicated that jujube and green tea could protect fibroblast cells against cytotoxic effects of UVB radiation and green tea exerted its protective effects at lower concentrations compared to jujube. 

Vayalil et al. (2004) ▶ showed that treatment with green tea as an antioxidant agent, could inhibit UVB-induced protein oxidation in HS68 fibroblast cells (Vayalil et al., 2004 ▶). Anggakusuma et al. (2010) ▶ also showed that treatment with macelignan as an antioxidant agent, could prevent UVB-induced cell death (Anggakusuma et al., 2010 ▶).

Kim et al. (2014) ▶ showed that ginseng leaves are rich in phenolic compounds and could prevent the formation of ROS species induced by UVA radiation (Kim et al., 2014 ▶).

To understand the mechanism behind these protective effects of jujube and green tea extract against UVB-induced cell damages, we evaluated the antioxidant capacity of the culture media when supplemented with different concentrations of these extracts. The results revealed that antioxidant capacity of green tea extract was about 8 times higher than that of jujube extract. This suggests that the antioxidant capacity of green tea may play an important role in its anti-UVB properties.

Plastina et al. (2012) ▶ showed that jujube is a precious fruit rich in bioactive and antioxidant compounds and beneficial to human health (Plastina et al., 2012 ▶). San et al. (2010) ▶ showed that leaves and fruits of jujube are good sources of phenolic compounds and the fruit is rich in catechin and rutin (San and Yildirim, 2010 ▶). Vahedi et al. (2008) ▶ showed that jujube could decrease the growth of human tumor cell lines and has a cytotoxic activity in them (Vahedi et al., 2008 ▶). Hoshyar et al. (2015) ▶ showed that jujube has a cytotoxic activity in cervical cancer cells (Hoshyar et al., 2015 ▶).

Brown (1999) ▶ conducted a systematic review on the properties of green tea. The results showed that green tea has a high polyphenolic content with antioxidant, anticarcinogenic, antimutagenic and antipromotional effects (Brown, 1999 ▶).

Nichols and Katiyar (2010) ▶ in a review article summarized the photoprotective effects of some selected polyphenols, such as green tea polyphenols on UV-induced skin inflammation and oxidative stress. The results showed that green tea has antioxidant, anti-inflammatory and anti-DNA damaging effects (Nichols and Katiyar, 2010 ▶).

However, we showed that both extracts not only augmented the antioxidant power of the culture media but also sustained the media for a day. Therefore, it could be concluded that jujube and green tea extracts empower the antioxidant capacity of cell culture media and consequently, protect fibroblast cells from oxidative stress-induced by UVB irradiation and increase cell viability.

Long-time exposure of the skin to UVB radiation causes changes in the extracellular matrix. MMP-2 and MMP-9 are matrix metalloproteinase that have important roles in photo-aging of the skin (Zaid et al., 2007 ▶). To determine if these MMPs are UVB inducible, the fibroblast cells were exposed to UVB radiation at 4mJ/cm^2 ^and/or jujube and green tea extract at different concentrations and then, incubated overnight at 37°C. The protein level of MMP-2 and MMP-9 were detected in the cell supernatants obtained from the above-noted cells. The result showed that UVB radiation can increase MMP-2 and MMP-9 in the culture of fibroblast cells exposed to UVB alone. Jujube and green tea extracts significantly decreased the protein level of MMP-2 and MMP-9 induced by UVB radiation. It was suggested that jujube and green tea extracts contain antioxidants (Hoshyar et al., 2015 ▶; Benelliet al., 2002 ▶). 

Because green tea polyphenols prevent other cutaneous adverse effects of UV, Vayalil et al. (2004) ▶ hypothesized that UV damages in mouse skin might be prevented by oral administration of green tea polyphenols* in vivo*. They also showed inhibition of UVB-induced protein oxidation in mouse model *in vivo* and in human skin fibroblast HS68 cell* in vitro*. Furthermore, oral administration of green tea polyphenols also resulted in inhibition of UVB-induced expression of MMP-2, -3, -7 and -9 in hairless mouse skin (Vayalil et al., 2004 ▶). Anggakusuma et al. (2010) ▶ evaluated the protective effects of macelignan in immortalized human keratinocytes (HaCaT) against UVB damage. Treatment with macelignan resulted in increased viability of HaCaT cells following UVB irradiation and inhibited MMP-9 secretion expression (Anggakusuma et al., 2010 ▶).

In conclusion, the results of this study showed that fibroblast cells viability was decreased when exposed to UVB radiation, however, the jujube and green tea extracts, significantly protected the cells against UVB radiation compared to control cells. Also, we suggested the antioxidant activity of jujube and green tea extracts as a potential determining in decreasing MMP-2 and MMP-9 induced by UVB radiation. 
